# Herpes Simplex Virus Encephalitis in a Post-operative Case of Follicular Thyroid Carcinoma

**DOI:** 10.15190/d.2024.13

**Published:** 2024-09-30

**Authors:** Anuragani Verma, Shruti Radera, Amita Jain, Nandini Mishra, Sheetal Agarwal, Om Prakash

**Affiliations:** ^1^Department of Microbiology, King George’s Medical University Lucknow, India

**Keywords:** Herpes simplex virus type 1, encephalitis, Tzanck smears

## Abstract

Herpes simplex virus (HSV) encephalitis is a life-threatening consequence of HSV infection of the central nervous system. Early antiviral therapy is most effective, necessitating prompt diagnosis. We report a case of atypical HSV encephalitis. The appearance of a strong headache followed by impairment of consciousness during the postoperative course in a 70-year-old patient who underwent surgical removal of a follicular thyroid carcinoma. Diffusion-weighted MRI detected brain abnormalities on the second day after the onset of symptoms, and polymerase chain reaction identification of HSV-1 DNA confirmed the diagnosis. A positive prognosis was achieved due to the decision to start specific, high-dose antiviral therapy based on clinical suspicion. A firm diagnosis was established by Tzanck smear and polymerase chain reaction.

## INTRODUCTION

Herpes simplex virus type 1 (HSV-1) is an enveloped, double-stranded linear DNA virus responsible for various human diseases, ranging from mucocutaneous, oral, and genital lesions to fulminant encephalitis^1^. The types of diseases caused by HSV-1 in patients depend on the route of infection and individual host factors. Importantly, this virus elicits lifelong infections by remaining dormant in neurons, from which rare viral reactivations may occur^2^. HSV-1 infection is common in cancer patients, resulting from the reactivation of the latent virus. HSV encephalitis (HSE) is the most common fatal sporadic encephalitis in humans^3^. About 90% of all HSE cases in adults are due to HSV-1^4^. Report a case of follicular carcinoma of the thyroid that showed HSV-1 infection with multinucleated cells on a Tzanck smear.

## CASE PRESENTATION

**Figure 1:**
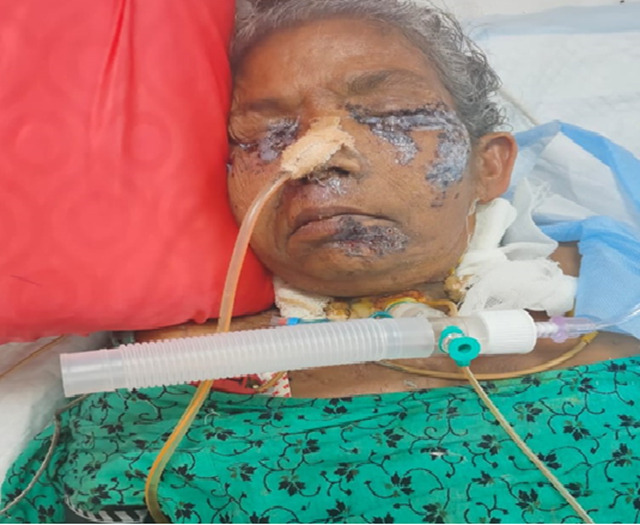
Multiple crusted lesions around the bilateral eyes and perioral location

**Figure 2:**
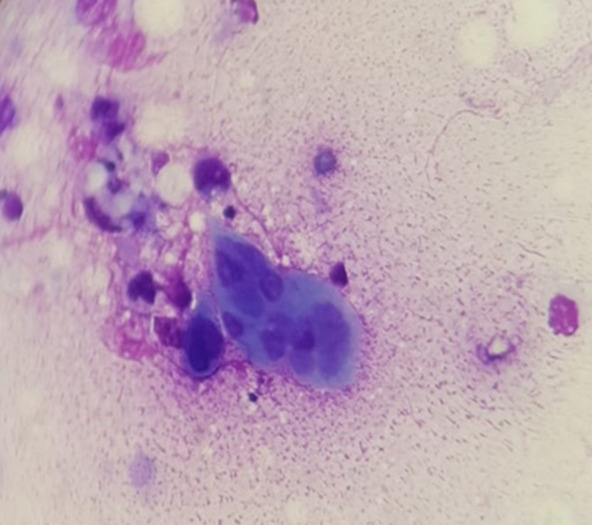
Photomicrograph of Tzanck smear from the perioral skin lesion showing multinucleated giant cells

A 70-year-old patient was admitted, evaluated for her complaints, and diagnosed with a case of follicular carcinoma of the thyroid (PT3AN0MX). She underwent a total thyroidectomy on March 23. The intraoperative period was uneventful, and the patient was managed with broad-spectrum antibiotics and electrolytes. Immediately after surgery, a 4/6 (house-Brackman grading) degree of facial palsy was evident. On postoperative Day 1 (POD-1) the patient developed seizures that may be secondary to dyselectrolytemia. A neurology opinion was sought for the same and electrolytes were corrected with 3% NaCl along with KCl according to neurology advice. On the second postoperative day, the patient reported moderate headache and asthenia and his body temperature was 37°C. She developed hypercalcemia and stridor. Infusion with calcium gluconate was started along with oral calcium supplementation. She was intubated and moved to the ICU for additional treatment because of her low GCS. She underwent a tracheostomy given her prolonged need for ventilatory support. Blood count showed neutrophilic leucocytosis (23200 cells/ml) and antibiotic therapy was continued without change. On the seventh postoperative day, the patient complained of a significant worsening of his headache, and multiple small fluid-filled lesions were noticed on her face around the mouth and eyes. The lesions were initially fluid-filled blisters, which later oozed fluid, formed a crust, and spread to the left upper arm (Figure 1). The patient continued to have low consciousness levels and was provisionally diagnosed with a case of herpes encephalitis. For the diagnosis, virological and radiological testing (CT and MRI brain scans) were done. T2-weighted magnetic resonance imaging of the brain showed a high-intensity area in the bilateral temporal lobes and insular cortex and the patient was radiologically diagnosed with herpetic encephalitis. Real-Time Polymerase Chain Reaction (RT-PCR) from serum, nasal swabs, lesions, and CSF was positive for HSV1 (Table 1). Tzanck smears showed multinucleated giant cells that are suggestive of HSV (Figure 2). Intravenous administration of acyclovir (750 mg TDS) for 3 weeks led to gradual improvement of consciousness and the patient was able to respond to verbal clues. Tzanck smears showed multinucleated giant cells that are suggestive of HSV (Figure 2). Intravenous administration of acyclovir (750 mg TDS) for 3 weeks led to gradual improvement of consciousness and the patient was able to respond to verbal clues.

**Table 1:** PCR test results for various viruses in different sample types

PCR test for	CSF	Serum	Lesion scrap	Nasal swab
Herpes simplex virus type 1 (HSV1)	Detected	Detected	Detected	Detected
Herpes simplex virus type 2 (HSV 2)	Not Detected	Not Detected	Not Detected	Not Detected
Varicella zoster virus (VZV)	Not Detected	Not Detected	Not Detected	Not Detected
Cytomegalovirus (CMV)	Not Detected	Not Detected	Not Detected	Not Detected

## DISCUSSION

HSV encephalitis (HSE) has an extremely high mortality rate of about 70%, with fewer than 30% of survivors returning to normal function^5^. The neuropathogenesis of HSE has been associated with the presence of latent HSV-1 in ganglionic tissues in healthy people and the tendency of the disease process to localize to the frontotemporal region of the brain^6,7^. Several circumstances have been incriminated as factors that possibly favour reactivation of a viral infection: receipt of steroids, receipt of radiotherapy, trauma, immune suppression, and stress. The common signs and symptoms of herpetic encephalitis are aphasia or mutism, personality change, focal or generalized seizures, and, in some cases, coma^7^. In this case, the patient had seizures, stridor, headache, and asthenia that were suggestive of CNS involvement. Examination of the cerebrospinal fluid (CSF) is of considerable diagnostic value in HSE and should always be performed after computed tomography or MRI.

Herpes simplex encephalitis (HSV) is a serious illness with an unacceptably high mortality rate. CSF polymerase chain reaction (PCR) for HSV DNA has been a major diagnostic advance and has helped to identify different patterns of HSV infection. PCR is used to make a definitive diagnosis of HSE^7,8^. In this patient, a diagnosis of HSV encephalitis was hypothesized, but a definitive diagnosis could not be made because all possible samples were positive for HSV1 by RTPCR and Tzanck smear. The advent of acyclovir has dramatically improved the mortality and morbidity of patients with HSE, especially if it is initiated early. It selectively inhibits the HSV-specific DNA polymerase and is activated specifically in HSV-infected cells^9,10^. The standard dose of acyclovir is 10 mg/kg three times daily for 14 days. In proven HSE, the duration of treatment can be extended to 21 days to prevent a relapse^11,12^. In this case, early treatment based upon standard guidelines could be started due to early diagnosis of HSV by RT PCR and Tzanck smear, which led to a better prognosis for the patient. However, HSE still has an unacceptably high mortality and morbidity. A better outcome is seen in early diagnosis and early treatment.

## CONCLUSION

An important clinical scenario is the reactivation of HSV-1 encephalitis in the post-operative period of thyroid cancer surgery, which occurs only in extreme cases. This instance demonstrates the importance of maintaining vigilance in cancer patients, even after medical treatment has been done. PCR testing, Tzanck smear, and diffusion-weighted magnetic resonance imaging were the three diagnostic methods that were utilized in this particular instance. Consequently, this resulted in an early diagnosis and the beginning of high-dose antiviral therapy, which improved the outcomes of an illness that had the potential to be lethal. The incident demonstrates the significance of early suspicion and involvement in cases that are comparable to this one.
